# Responsiveness and Precision of Digital IMUs under Linear and Curvilinear Motion Conditions for Local Navigation and Positioning in Advanced Smart Mobility

**DOI:** 10.3390/mi15060727

**Published:** 2024-05-30

**Authors:** Luciano Chiominto, Emanuela Natale, Giulio D’Emilia, Sante Alessandro Grieco, Andrea Prato, Alessio Facello, Alessandro Schiavi

**Affiliations:** 1 Department of Industrial and Information Engineering and Economics, University of L’Aquila, 67100 L’Aquila, Italy; luciano.chiominto@graduate.univaq.it (L.C.); emanuela.natale@univaq.it (E.N.); giulio.demilia@univaq.it (G.D.); 2 Department of Engineering “Enzo Ferrari”, University of Modena and Reggio Emilia, 41125 Modena, Italy; santealessandro.grieco@studenti.polito.it; 3 Division of Applied Metrology and Engineering INRiM—National Institute of Metrological Research, 10135 Turin, Italy; a.facello@inrim.it (A.F.); a.schiavi@inrim.it (A.S.)

**Keywords:** digital IMU, precision, curvilinear motion, in-lab calibration

## Abstract

Sensors based on MEMS technology, in particular Inertial Measurement Units (IMUs), when installed on vehicles, provide a real-time full estimation of vehicles’ state vector (e.g., position, velocity, yaw angle, angular rate, acceleration), which is required for the planning and control of cars’ trajectories, as well as managing the in-car local navigation and positioning tasks. Moreover, data provided by the IMUs, integrated with the data of multiple inputs from other sensing systems (such as Lidar, cameras, and GPS) within the vehicle, and with the surrounding information exchanged in real time (vehicle to vehicle, vehicle to infrastructure, or vehicle to other entities), can be exploited to actualize the full implementation of “smart mobility” on a large scale. On the other hand, “smart mobility” (which is expected to improve road safety, reduce traffic congestion and environmental burden, and enhance the sustainability of mobility as a whole), to be safe and functional on a large scale, should be supported by highly accurate and trustworthy technologies based on precise and reliable sensors and systems. It is known that the accuracy and precision of data supplied by appropriately in-lab-calibrated IMUs (with respect to the primary or secondary standard in order to provide traceability to the International System of Units) allow guaranteeing high quality, reliable information managed by processing systems, since they are reproducible, repeatable, and traceable. In this work, the effective responsiveness and the related precision of digital IMUs, under sinusoidal linear and curvilinear motion conditions at 5 Hz, 10 Hz, and 20 Hz, are investigated on the basis of metrological approaches in laboratory standard conditions only. As a first step, in-lab calibrations allow one to reduce the variables of uncontrolled boundary conditions (e.g., occurring in vehicles in on-site tests) in order to identify the IMUs’ sensitivity in a stable and reproducible environment. For this purpose, a new calibration system, based on an oscillating rotating table was developed to reproduce the dynamic conditions of use in the field, and the results are compared with calibration data obtained on linear calibration benches.

## 1. Introduction

The new generation of vehicles is currently able to supply advanced assistance to the driver in their driving tasks [1,2,3] by playing an active role in safety issues, such as electronic stability control (ESC) [4], antilock braking (ABS) [5], and the newer Advanced Driver Assistance Systems (ADAS) [6,7,8], as well as by managing in-car navigation and positioning technologies based on Inertial Measurement Units (IMUs), Global Positioning Systems (GPSs), and map matching [9,10,11]. Moreover, vehicles are already connected [12,13,14], since they can link with smartphones, providing emergency roadside assistance, real-time traffic alerts, and circulation [15,16,17,18], laying the basis for the effective and functional development of Intelligent Transport Systems (ITSs), or “smart mobility” [19,20].

In particular, IMUs, based on MEMS technology, have become essentials in smart mobility development by giving great advantages over conventional sensors and other technologies due to their low cost, light weight, and low power consumption; their array of applications is expanding, and they will play an important role in supporting the evolution of ADAS [21] and the development of full Autonomous Vehicles (AVs) [22,23]. Some studies estimate that AVs could significantly reduce energy use by up to ~80% once platooning, parking, and automated ridesharing [24] are effectively and safely actualized and largely diffused.

Nevertheless, there is still a lack of technical standardization for IMU MEMS-based sensors [25,26] to supply a full metrological characterization, a quantification of reliability, and an adequate performance compliance assessment, resulting in an overall loss of safety and reliability of the systems through which these sensors are exploited. Despite the huge technical and scientific literature issued in the past decades related to the mechanical testing of automotive IMUs in many different working conditions (for a comprehensive survey, readers can rely on [27,28,29,30,31,32,33,34] and related references), fewer studies are available on metrological approaches (in controlled standard laboratory conditions) and interlaboratory comparisons to validate experimental procedures, evidence, and results [35,36,37]. The importance of providing suitable test procedures under controlled laboratory conditions, based on repeatable and reproducible methods, is supposed to be a fundamental basis for the development of ad hoc standards, as well as a reference for the realization of new calibration and test systems linking primary laboratories (National Metrology Institutes) to the metrological chain, as well as accredited calibration laboratories to repair workshops for cars for the implementation of periodic control systems on-site.

Recently, metrological approaches to provide a suitable characterization of IMU MEMS-based sensors’ technical performance in static and dynamic working conditions were investigated and applied [4,38,39]. It has been demonstrated that the identification and the quantification of fundamental metrological attributes, based on calibration procedures such as accuracy, precision, uncertainties budgets, coverage factors, reproducibility, conformity among sensors, and traceability, allows for improving the overall trustworthiness and quality of the data that are supplied, managed, and processed [40,41,42]. Indeed, if IMUs provide effectively comparable and reliable data, the information managed and exchanged in operating conditions is trustworthy and safer, as discussed in Section 2.

## 2. Local Navigation and Positioning Systems: A Brief Survey

**Figure 1:**
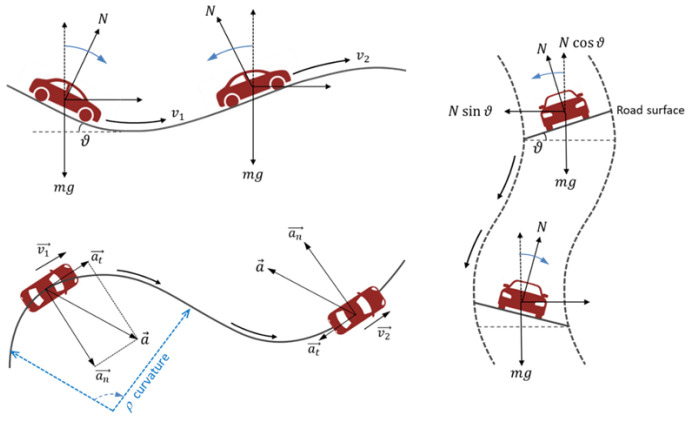
Illustrative examples of curvilinear motions (vertical, horizontal, and lateral) that occur in a moving vehicle.

A highly reliable and precise local navigation and positioning system is important for the safe and functional development of automation in vehicles. From this perspective, the focus of this work is to provide an in-lab metrological characterization of IMU MEMS-based sensors integrated into vehicles to support local navigation and positioning tasks in peculiar working conditions, such as in curvilinear motions. As it is known, the trajectory of a moving vehicle is subjected to continuous more or less sudden changes in direction, including horizontal (e.g., a tortuous road), vertical (e.g., dips and humps in the road), and lateral ones (e.g., a circular banked road), as illustratively shown in Figure 1.

**Figure 2:**
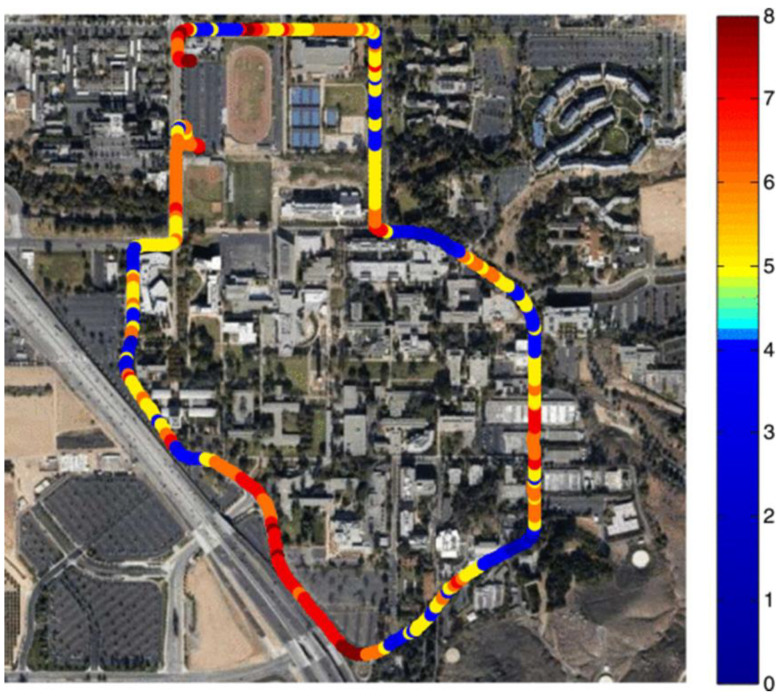
Test trajectory is marked with colors to represent the number of satellites available to the receiver along the trajectory [43].

For such applications that require trajectories for the planning and control of a vehicle’s stability conditions, the estimation of the full vehicle state vector (e.g., position, velocity, yaw angle, angular rate, acceleration) is essential. Usually, new vehicles use Global Positioning System (GPS) receiver sensors to establish their global navigation and positioning, and the required maneuvers are performed with respect to a digital map referenced in a standard geodetic frame. Usually, a well-designed GPS unit receives at least four satellite signals to achieve 3 m to 8 m positioning accuracy [43]. On the other hand, the use of GPS alone has certain limitations. In fact, in urban environments, GPS signals can be blocked by trees or tall buildings, and their use alone is not sufficient to determine the local positioning of a vehicle. Moreover, GPSs cannot provide accurate, high-bandwidth estimates of the full vehicle state vector. Figure 2 shows typical GPS coverage in an urban environment, resulting in the use of multiple GPS satellites to receive a sufficient signal.

These quantities can be sensed by MEMS, resulting in a reduction in cost since MEMS IMUs are becoming much cheaper than optical or radar sensors that could perform the same task but with a different approach. In the work in [44], it is shown that an IMU integrated with a GNSS (Global Navigation Satellite System) of a vehicle is able to substitute the tasks performed by the latter whenever the signals coming from the satellites are not available. In particular, the redundancy of MEMS sensors has shown better performances in filling the gaps in the GNSS by providing a continuous navigation and positioning signal under severe environmental conditions. As a consequence, it is important to investigate the actual responsiveness and precision of the MEMS sensor accelerometer when subjected to several sudden trajectory variations, characterized by different linear, tangential, and centripetal accelerations. The metrological characterization of the performance of IMU sensors, in static or linear dynamic conditions (as previously studied), may not be representative and accurate enough compared with curvilinear dynamic working conditions with respect to road geometry [45,46,47]. At present, the sensors and algorithms of AVs are still having trouble identifying road hazards and potential obstacles reasonably expected to be in the driving path [48], and at present, the legal framework has many regulatory gaps that have to be filled, so it is necessary to provide practical evidence and technical procedures to develop a suitable standardization supporting the manufacturers to ensure that AVs and integrated sensors work correctly [49].

With a proper experimental setup and specific devices, it has been possible to analyze and evaluate how the accuracy and precision of the data provided by the accelerometer integrated into the IMUs change according to different scenarios and operating modes. The IMUs are subjected to both sinusoidal linear and sinusoidal curvilinear motions, and the responsiveness, in terms of IMUs sensitivity with respect to linear, tangential, and centripetal accelerations, is investigated and compared. The experimental work is carried out in environment-controlled standard laboratory conditions on three MEMS belonging to the same production line batch in order to have a complete overview of the functioning from a metrological point of view.

## 3. Materials and Method

### 3.1. The IMUs and the Supporting External Microcontroller

**Figure 3:**
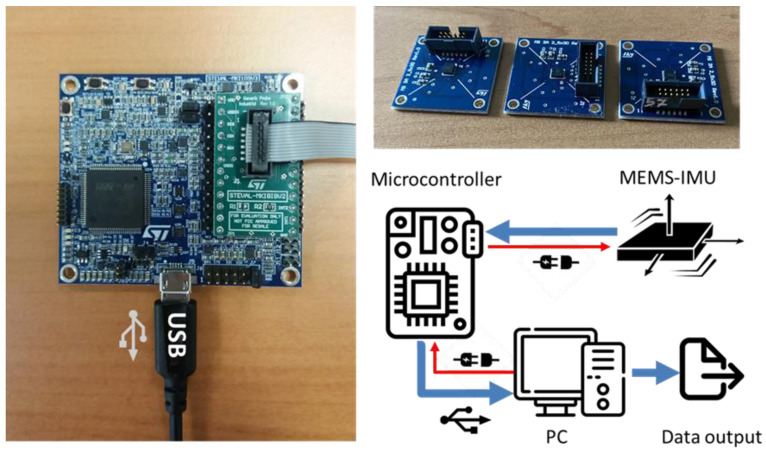
The set of 3 digital 3-axis MEMS accelerometers, the microcontroller, and the schematic representation of the sensing system under investigation.

In this study, we examine three commercial, low-power digital MEMS-based Inertial Measurement Units (referred to in the following as “MEMS 1”, “MEMS 2”, and “MEMS 3”) manufactured by STMicroelectronics, specifically the LSM6DSR model [50]. Each IMU, belonging to the same production line batch, consists of a 3D accelerometer, a 3D gyroscope, a charge amplifier, and an analog-to-digital converter. For the purposes of this investigation, the 3D gyroscope is not utilized and remains turned off, since only sensitivity related to acceleration is investigated. The digital MEMS accelerometers are connected to an external microcontroller by STMicroelectronics (Agrate Brianza, MB, Italy) model STEVAL MKIGIBV2 [51] through a serial cable. This microcontroller acquires the digital sensor data, communicating with a PC via a USB cable, as schematically shown in Figure 3.

The signal is acquired by means of a Serial Peripheral Interface (SPI), which is a synchronous serial communication interface used for connecting digital sensors. The 1-bit signal from the ΣΔ-ADC is then converted through a decimation process and a low-pass filter into a standard 16-bit-signed PCM (Pulse Code Modulation) signal, with a nominal sampling frequency rate of 1660 Hz. According to the manufacturer [50], however, sampling frequencies up to −6% of the target, i.e., up to 1560 Hz, can be expected. For this reason, the actual sampling frequency of every MEMS is previously evaluated by counting the number of samples of the known generated sinusoidal signals. Sampling frequencies range from around 1580 Hz to 1630 Hz. Amplitude values range between −2^16-1^ = −32,768 D_16-bit-signed_ and +(2^16-1^ − 1) = +32,767 D_16-bit-signed_, where the digit unit is a signed 16-bit sequence converted into a decimal number.

The linear acceleration sensitivity of a digital MEMS accelerometer, expressed by the manufacturer in terms of mg/LSB (Least Significant Bit), depends on the “full scale” used in the testing condition, and it is conventionally attributed to every sensitive axis of the sensor for static and dynamic measurements, independently from frequency, without any indication of the associated uncertainty, and it is not evaluated through traceable calibration methods. In this work, by using a “full scale” of ±8 g, the sensitivity declared is 0.224 mg/LSB. In decimal units, it corresponds to 0.244 mg/D_16-bit-signed_, i.e., 2.39 × 10^−3^ (m/s^2^)/D_16-bit-signed_. As for analog transducers, the metrological sensitivity is expressed as a function of the reference quantity, thus corresponding to 418 D_16-bit-signed_/(m/s^2^). By “metrological sensitivity”, we mean the quantitative ratio between the digital output (provided by the sensor in calibration) and the physical input (a frequency–amplitude-controlled reference vibration).

### 3.2. Metrological Characterization Methods

The proposed metrological characterization method provides the determination of the response, in terms of precision and accuracy, of the IMU’s MEMS accelerometer sensor with respect to both linear and curvilinear motions along the three sensitive axes. As a first step, it is necessary to evaluate the linear response of the sensor compared with a reference standard acceleration. To determine the metrological sensitivity of the sensor with respect to linear acceleration, a calibration is carried out using a tilt plate as a support, as described in detail in previous works [52,53]. The calibration system and procedure, developed at INRiM, allow one to provide the sensor sensitivity according to ISO 16063-21 [54] and thus the traceability to SI. Once the response and precision of the investigated sensors are known with respect to linear dynamic motions in a certain frequency range of interest, the responsiveness and the precision with respect to curvilinear dynamic motions are investigated. Results are then compared and discussed.

The responsiveness of DUTs subjected to curvilinear dynamic motions can be investigated on the basis of existing protocols already developed in applied metrology. Namely, ISO Standard 16063-15 [55] specifies the instrumentation and procedures used for the primary angular vibration calibration of angular transducers, i.e., angular accelerometers, angular velocity transducers, and rotational angle transducers to provide the traceability to SI. The systems used to generate angular vibration exciters are based on a brushless electric motor, and they are also called “rate tables” and are commonly applied for testing inertial navigation sensors. Moreover, recent studies investigated the response of DUTs under constant rotation rate [56,57,58], as well as for high-resolution accelerometers [59], producing a time-varying sinusoidal excitation in the earth’s gravitational field to provide a calibration method for a 3-axis accelerometer and the investigation of the related intrinsic properties.

In the present work, the method applied to generate sinusoidal curvilinear motion is based on the same principle; nevertheless, the novelty of the calibration is to provide simultaneous responsiveness of IMUs with respect to tangential and centripetal acceleration variations by applying a variable rotation rate by using a proper calibration system developed at the University of L’Aquila. Indeed, an oscillating motion of the “rate table” is generated with sequential changes in direction, which involves controlled variations in both the angular and tangential velocity and the centripetal and tangential acceleration.

Moreover, it should be noted that potential variables that could influence sensitivity measurements were previously investigated [60]: the thermal effects on sensors are considered negligible, at least in the range from −20 °C to 80 °C, as well as the stress/fatigue effects due to high vibration amplitude levels or shocks. This evidence allows one to guarantee the stability of the investigated DUTs in operating conditions, although more severe analysis based on accelerated aging methods (not investigated here) could provide different results related to long-term stability.

### 3.3. Linear Dynamic Motions Characterization

**Figure 4:**
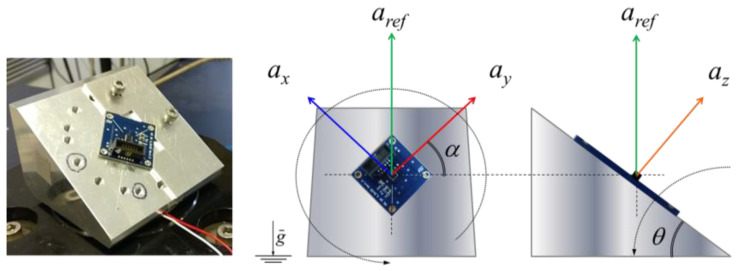
The multicomponent calibration system. The inclined plane allows one to generate a projection of the vertical reference acceleration along three axes simultaneously.

The experimental set-up allows one to generate a projection of the vertical reference acceleration, *a_ref_*, along three axes simultaneously. The calibration method and the experimental set-up, shown in Figure 4, are described in detail in [52,53].

By means of simple geometrical laws, it is possible to define the expected acceleration along the three main axes as follows:

(1) $$\left\{\begin{array}{c} a_{x}=\left|a_{ref}\sin\left(\theta \right)\cos\left(\alpha \right)\right|\\ a_{y}=\left|a_{ref}\sin\left(\theta \right)\sin\left(\alpha \right)\right|\\ a_{z}=\left|a_{ref}\cos\left(\theta \right)\right| \end{array}\right.,$$

where θ is the tilt angle (tolerance of 0.1°), and α is the rotation angle (tolerance of 0.1°). Calibration is performed at several different tilt and rotation angles in order to avoid correlation between the independent variables θ and *α*, as well as with *a_ref_*, which is constant. This procedure is detailed and described in [55].

#### 3.3.1. Test Plan

The calibration is carried out at 3 vertical oscillation frequencies: 5 Hz, 10 Hz, and 20 Hz. The amplitude of the vertical reference acceleration, *a_ref_*, is set at 10 ms^−2^.

Measurements are carried out in four configurations, achieved by fixing the MEMS accelerometer to the center of the vibrating table using ultrathin double-sided adhesive tape at various angles of tilt and rotation, specifically a tilt of 0° and rotation of 270°, a tilt of 15° and rotation of 90°, a tilt of 75° and rotation of 0°, and a tilt of 75° and rotation of 90°. Values of sensitivity, along the MEMS axes, are determined as the average values from 4 repetitions in different configurations; the associated uncertainties are composed of the variance among results and instrumental uncertainties.

#### 3.3.2. Data Processing Procedure

Acceleration along the vertical axis, namely $a_{ref}$, is determined using a single-axis reference transducer (integrated within the stroke of the shaker) calibrated against the INRiM primary standard. This measurement is collected through the NI 4431 acquisition board integrated into the PC, with a sampling rate of 50 kHz and processed via LabVIEW^®^ 2021 software to yield the RMS reference value in m s^−2^. The external microcontroller acquires the digital MEMS output at a maximum sampling rate of 6.660 kHz, saving the data as binary files. These files are then subjected to processing in MATLAB^®^ 2022a software: the RMS digital value for each specific frequency *f*, expressed in Decimal_16-bit-signed_ format, is derived by applying a first-order Butterworth band-pass filter centered at the frequency of interest with a fractional bandwidth of 10% to the temporal digital signals. Subsequently, the Root Mean Square is computed to eliminate the offset resulting from gravity and the impact of background vibrations.

### 3.4. Curvilinear Dynamic Motions Characterization

**Figure 5:**
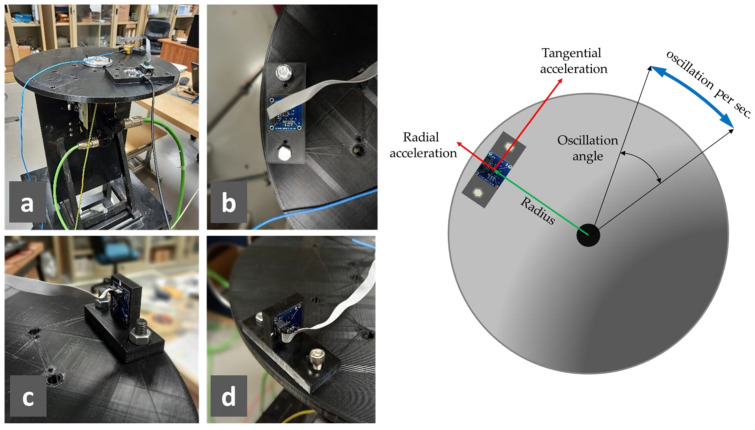
Pictures of the rotating test bench: (**a**) lateral view (the support for the microcontroller is visible); (**b**) support to orient the X- and Y-axis in the radial or tangential direction; (**c**) support to orient the Z-axis in the radial direction; (**d**) support to orient the Z-axis in the tangential direction. Schematic representation of the rotating test bench functioning.

The three MEMS sensors under test are calibrated on a 3D-printed PLA (poly lactic acid) disk, rotating around its symmetry axis, and mounted on a servomotor’s axis (Figure 5a). Specific supports, also in PLA, were created to fix the accelerometers and the microcontroller on the disk, in particular, the following ones:

- One which allows the X- and Y-axis to be oriented in the radial or tangential direction, and the Z-axis vertically (Figure 5b);
- One which allows the Z-axis to be oriented in the radial direction (Figure 5c);
- One which allows the Z-axis to be oriented in the tangential direction (Figure 5d);
- One for attaching the microcontroller (Figure 5a).

Since the frequency of the radial acceleration is double that of the tangential one, as is explained later, the correct positioning of the accelerometers was checked by verifying that any radial component of the tangential acceleration was negligible.

The disk rotation is managed by a servomotor manufactured by Schneider Electric by means of a high-precision angular encoder that is connected to a programmable logic controller (PLC), enabling the realization of a sinusoidal motion law for the angular position (and therefore also for velocity and acceleration). To realize the sinusoidal movement, the inputs of the PLC are oscillation angle and frequency.

The control program has access to encoder data at a sampling frequency of 1000 Hz. Then, the data generated are stored as a binary file using the built-in “TraceSyn” tool of the Schneider Electric software SoMachine Motion builder 4.31.

In addition to the measured angular position $\theta \left(t\right)=\theta_{0}\cos\left(2\pi ft\right)$, the system also provides the angular speed $\omega \left(t\right)=2\pi f\theta_{0}\sin\left(2\pi ft\right)=\omega_{0}\sin\left(2\pi ft\right)$, obtained as a derivative of the first. This information was used to calculate the reference centripetal and tangential accelerations, as indicated in the following equations:

(2) $$a_{r}=r\omega^{2}\left(t\right)=r{\left(\omega_{0}\sin\left(2\pi ft\right)\right)}^{2}=r\frac{\omega_{0}^{2}}{2}\left(1-\cos\left(2\pi (2f)t\right)\right)$$

(3) $$a_{t}=r\frac{d\left(\omega \left(t\right)\right)}{dt}=2\pi fr\omega \left(t\right)=2\pi fr\omega_{0}\sin\left(2\pi ft\right)$$

where *f* is the oscillation frequency, and *r* is the distance of the sensitive element from the axis of rotation. The disk and supports are designed in such a way that, in all intended sensor positions, the center of the sensor is at a distance from the axis of rotation equal to *r* = 170 mm: this distance was also verified through a caliper with a resolution of 0.05 mm.

It should be noted that in Equation (2), a sinusoidal square function ($\omega^{2}(t)$) is present, and trigonometric formulas explain that it is characterized by double the frequency; for this reason, the radial acceleration has a double frequency compared with the tangential one.

**Figure 6:**
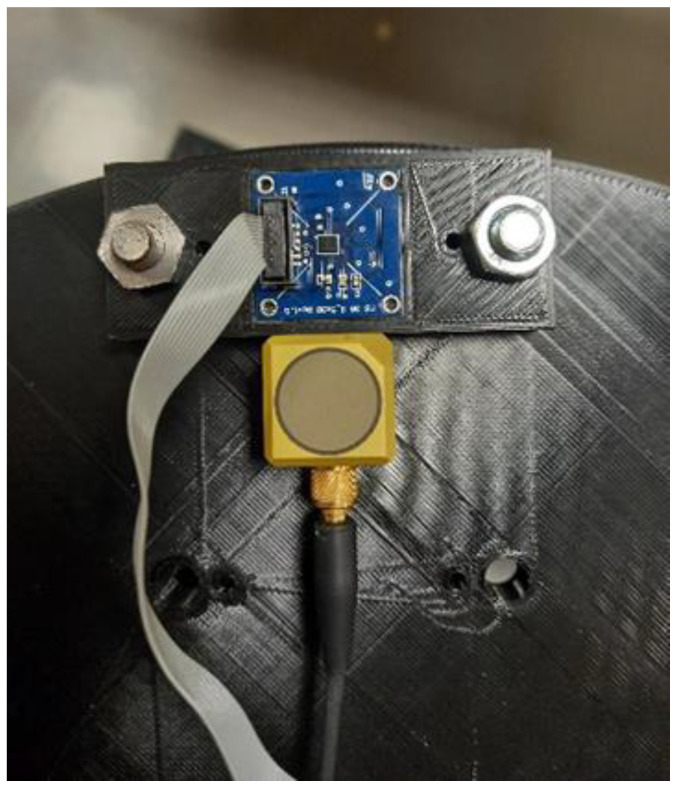
Details of the MEMS accelerometer under test and IEPE reference accelerometer.

In addition, a reference IEPE (integrated electronics piezoelectric) sensor (Model TLD356B18 by PCB Piezotronics (Depew, NY, USA), Measurement Range: ±5 g) was placed against the MEMS support, as indicated in Figure 6, using double-sided tape, always in such a way that one axis is radial and one tangential; the distance of the sensitive element from the rotation axis is measured the same way as the MEMS sensor, and the result is equal to 145 mm.

Reference accelerometer signals are acquired by a National Instruments (NI) CompactDAQ 9132 system and an NI 9234 input module for IEPE accelerometers. The acceleration values for the three axes of the IEPE accelerometer are acquired at a sampling rate of 1704 Hz and recorded using a LabVIEW program. A comparison between the two ways of evaluating the reference accelerations is also carried out.

#### 3.4.1. Test Plan

The tests are carried out at three oscillation frequencies: 5 Hz, 8 Hz, and 10 Hz.

The oscillation angles are selected in order to achieve the maximum acceleration without exceeding the ±5 g (g is the gravitational acceleration) limit of the IEPE accelerometer. In particular, the theoretical test conditions are as follows:

- A 5 Hz oscillation frequency and angle of 34°: In this configuration, the tangential and radial amplitude, at the radius of 170 mm, are 48.8 m/s^2^ and 14.8 m/s^2^, respectively;
- An 8 Hz oscillation frequency and angle of 13°: In this configuration, the tangential and radial amplitude, at the radius of 170 mm, are 48.7 m/s^2^ and 5.5 m/s^2^, respectively;
- A 10 Hz oscillation frequency and angle of 8.5°: In this configuration, the tangential and radial amplitude, at the radius of 170 mm, are 49.8 m/s^2^ and 3.7 m/s^2^, respectively.

For each configuration (angle and oscillation frequency), the MEMS sensors were analyzed in such a way as to stress all three axes, both in the radial and tangential direction, using the specifically made supports.

For each configuration and each position of the accelerometers, 10 repeated tests were carried out, and in each of them, the signals from the digital MEMS, the IEPE accelerometer, and the encoder were recorded for 40 s.

#### 3.4.2. Data Processing Procedure

The data processing and analysis consist of different steps, performed using MATLAB R2023b:

1. After importing the data, the IEPE acceleration values are converted into m s^−2^ using the calibration sensitivity of each axis. Then, both the signal from the digital MEMS and the IEPE reference accelerometer are filtered using a 6-pole digital low-pass Butterworth filter with a cutting frequency of 40 Hz.
2. The encoder data are processed as indicated in Equations (2) and (3) to obtain the reference tangential and radial accelerations.
3. A zero-crossing algorithm is implemented to select an entire number of signal periods to avoid the leakage phenomenon in further analysis.
4. As stated in a previous work by the authors [58], the sampling frequency of the digital MEMS is not constant, and the mean value is not equal to the selected one. For this reason, a procedure to evaluate the mean value of the sampling frequency is adopted as follows:
  - The signal is processed using a fast Fourier transform (FFT) at different sampling frequencies, ranging from 1560 Hz to 1760 Hz with a step of 1 Hz;
  - The mean sampling frequency is identified as the one that maximizes the amplitude at the oscillation frequency (5 Hz, 8 Hz, or 10 Hz in the trials carried out).

Finally, to evaluate the amplitude of the accelerations, each signal is processed using the FFT. For the tangential acceleration, the value of the component at the oscillation frequency is considered. For the radial acceleration, the component of interest corresponds to twice the oscillation frequency. For example, in the case of 5 Hz oscillation, the tangential acceleration has the same frequency, while the radial acceleration has a frequency of 10 Hz.

## 4. Experimental Results

### 4.1. Linear Dynamic Motions Responsiveness

**Figure 7:**
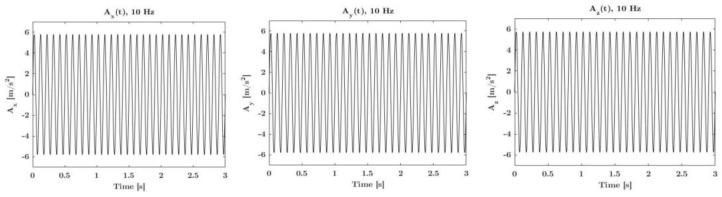
Actual real-time amplitude response along the *X*-axis, *Y*-axis, and *Z*-axis of the digital MEMS subjected to sinusoidal linear motions.

Experimental metrological sensitivities are determined by applying an almost constant peak amplitude of 10 m/s^2^ at 5 Hz, 10 Hz, and 20 Hz. These sensitivities are then compared with the expected acceleration values calculated from Equation (1) taking into account inclination and rotation angles. Any difference between the experimental acceleration values along the three axes and the expected values depends on potential variations in the sensitivities of the sensors and potential misalignment within them. Once these differences are identified, adjustments can be made to fine-tune the actual metrological sensitivities of the IMU. In Figure 7, the accelerations along the *X*-axis, *Y*-axis, and *Z*-axis, at 10 Hz, as a function of time, are shown.

**Figure 8:**
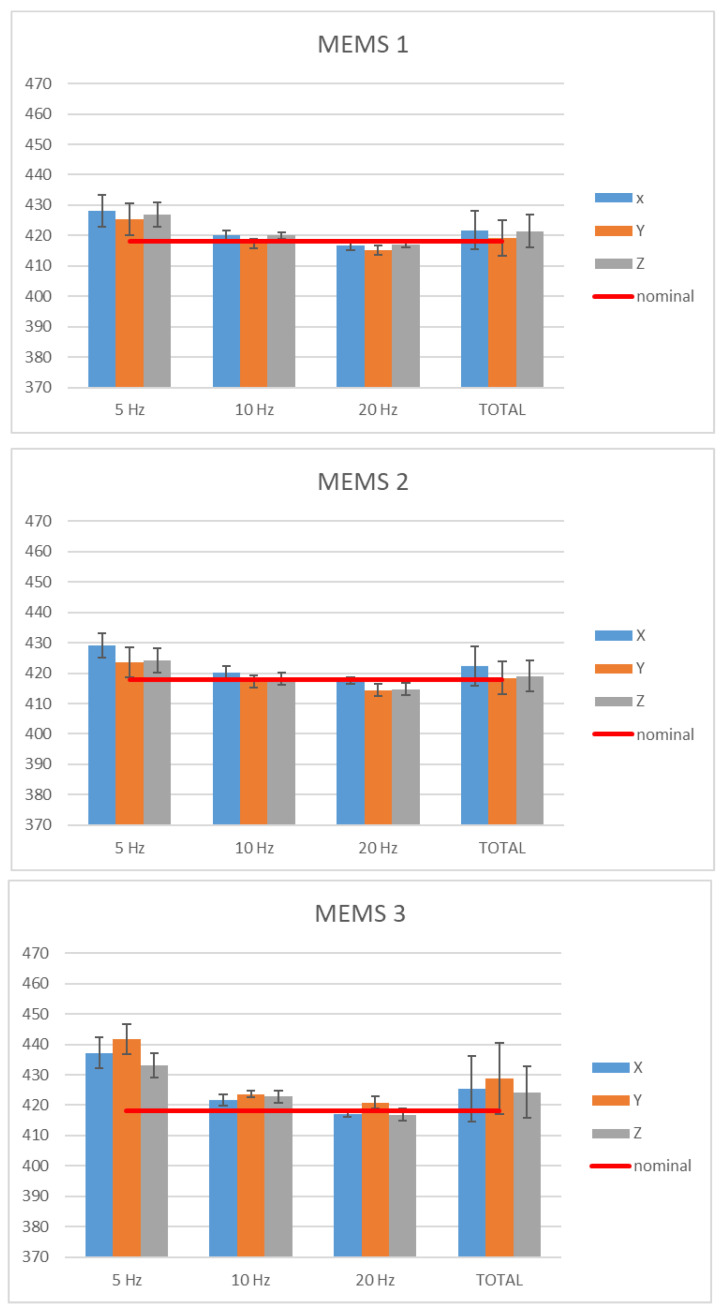
Sensitivities along the *X*-axis, *Y*-axis, and *Z*-axis of the digital MEMS subjected to linear motions.

In Figure 8, the sensitivity values (within uncertainties) along the *X*-axis, *Y*-axis, and *Z*-axis, at 5 Hz, 10 Hz, and 20 Hz, obtained from the calibration method described above, and the average values (total) are shown; the red line represents the nominal value provided by the manufacturer.

### 4.2. Curvilinear Dynamic Motions Responsiveness

**Figure 9:**
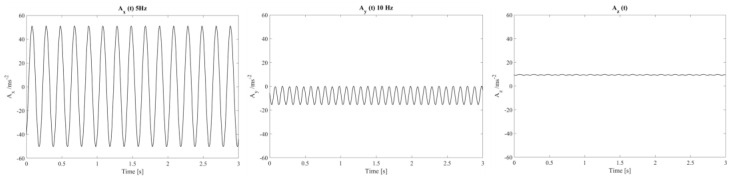
Actual real-time amplitude response along the *X*-axis, *Y*-axis, and *Z*-axis of the digital MEMS subjected to sinusoidal curvilinear dynamic motions.

Figure 9 presents the tangential acceleration along the *X*-axis and the radial acceleration along the *Y*-axis as a function of time. As shown in Figure 9, the tangential acceleration frequency is 5 Hz, as is the oscillation, while the radial acceleration frequency is 10 Hz.

In all the trials, the differences between the reference accelerations (by IEPE accelerometer and by encoder data) are not higher than 2%. Considering that the uncertainty of the IEPE declared by the manufacturer is 2%, the agreement between the two systems is considered satisfactory. Furthermore, traceability is given to the reference acceleration by the encoder system. The uncertainty of the accelerations calculated on the basis of the encoder data can be evaluated on the order of 0.5%, considering the uncertainties of radius *r* and angular position.

**Figure 10:**
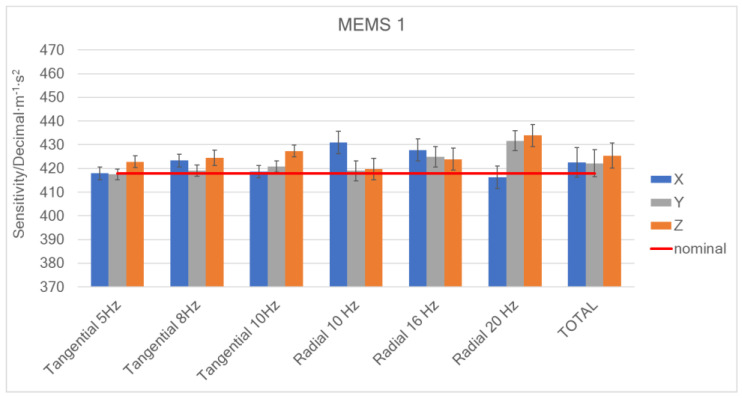

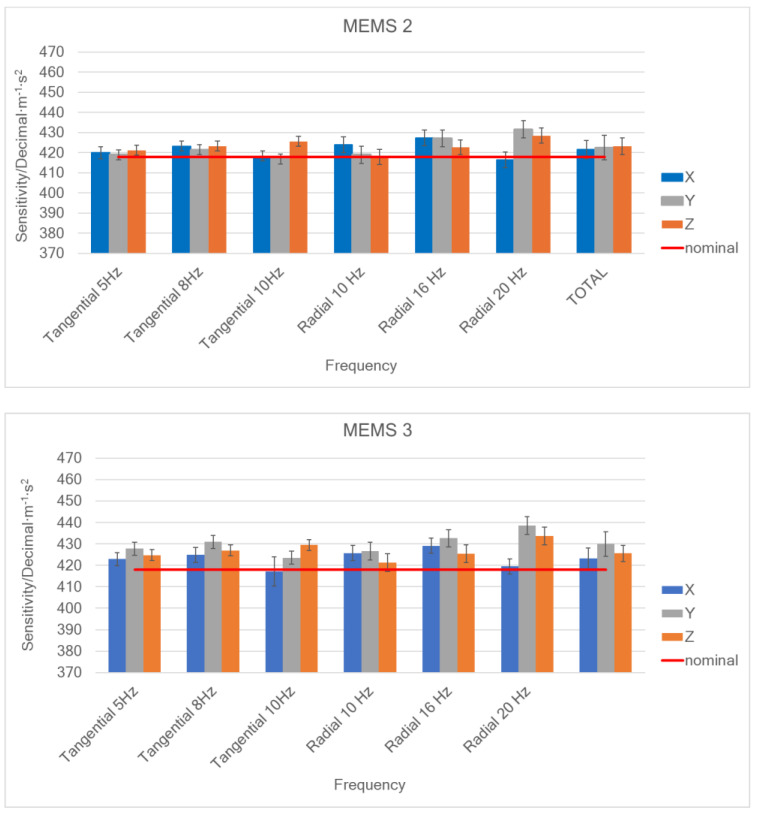
Sensitivities of the three digital MEMS considered.

Figure 10 shows the evaluated sensitivities considering the aforementioned test plan: error bars represent the uncertainty of the results, expressed as standard deviation, and obtained by combining repeatability contributions with bench uncertainty.

The sensitivity is represented considering the results corresponding to the two acceleration directions (“Tangential” and “Radial”) and the average of all the obtained values (“Total”). The acceleration values obtained on the basis of the encoder output were used as reference values for calculating the sensitivities. In Figure 10, the red line shows the sensitivity provided by the manufacturer’s datasheet.

It is noted that the variability, which in the case of sensitivities determined in the tangential direction is on average equal to 0.3%, increases to approximately 1% in the case of the radial ones. The reasons may lie in the smaller amplitude of the radial accelerations, which are placed lower in the measurement range of the accelerometers (both the MEMS and reference ones).

### 4.3. Experimental Comparison and Data Compatibility

In this analysis, comparisons are made to detect any significant differences between the following:

- Sensitivities determined on the rotating and linear bench;
- Sensitivities determined on the rotating bench and value provided by the manufacturer;
- Sensitivities of the *X*, *Y,* and *Z* axes;
- Sensitivities determined in radial and tangential directions;
- Sensitivities of the three accelerometers examined.

**Figure 11:**
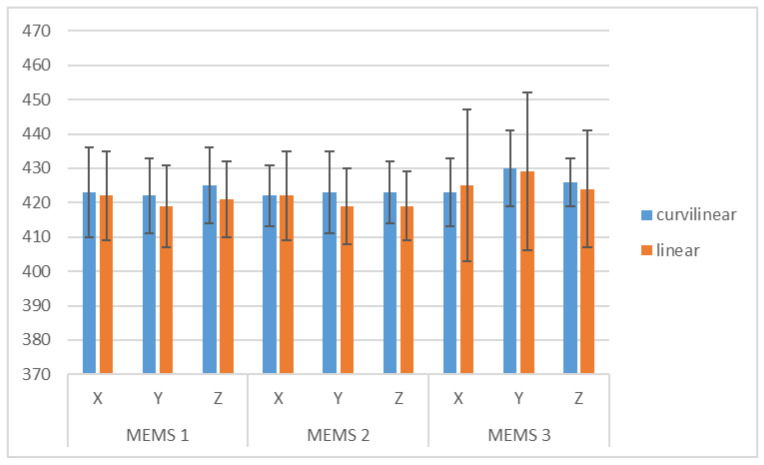
Comparison of the average sensitivities of the three digital MEMS determined from the sinusoidal linear (orange) and sinusoidal curvilinear (blue) dynamic characterization.

For the comparison between the results obtained on the linear and rotating bench, the average values for the sensitivities of the three axes in all the analysis conditions were calculated, as shown in Table 1 and in the graph of Figure 11. The overall uncertainty for each mean value was evaluated as the composition of the mean uncertainty from the single values and the standard deviation between the values over which the mean was calculated.

It can be observed that the uncertainties of the two benches are similar, except for the MEMS 3 accelerometer, for which the variability among the values obtained at different frequencies on the linear bench is higher.

These data were compared using the normalized error [40], according to ISO/IEC 17043:2023 [61].

In particular, for the comparison between two mean sensitivity values *S*_1_ and *S*_2_ from the two test benches of extended uncertainty *U*_1_ and *U*_2_, respectively, the normalized error *E*_n_ is calculated as follows:

(4) $$E_{n}=\frac{\left|S_{1}-S_{2}\right|}{\sqrt{U_{1}^{2}+U_{2}^{2}}}$$

Data can be considered compatible when *E*_n_ ≤ 1.

In Table 2, the normalized errors to compare results obtained on the linear and rotating bench are shown; in all cases *E*_n_ < 1, so the results are compatible.

Similarly, with respect to the sensitivity value provided by the manufacturer, which is unique to the three axes and equal to 418 D_16-bit-signed_/(m/s^2^) ± 1% of tolerance (standard deviation obtained by hypothesizing a rectangular distribution), no significant differences were detected for the sensitivities of the *X*, *Y,* and *Z* axes for all the examined accelerometers.

The comparison between the *X*, *Y,* and *Z* axes also provides compatible results, and no significant differences were detected between different frequencies, either in the case of radial or tangential accelerations.

Finally, no significant differences were detected between different accelerometers.

## 5. Discussion and Conclusions

The safe and functional development of automation in vehicles also relies on the accuracy and reliability of sensors managing and supporting advanced assistance to drivers in their driving tasks. However, at present, the legal framework related to sensors integrated into vehicles has many regulatory gaps, so it is necessary to provide practical evidence and technical procedures to develop a suitable standardization supporting the manufacturers to ensure the trustworthiness of the sensors and data provided based on recognized, agreed upon, and replicable protocols. From this perspective, this paper proposed two technical protocols (that can be replicated for proficiency tests in laboratories) to investigate and compare the performance of Inertial Measurement Units (IUMs) in terms of responsiveness and precision under linear and curvilinear motion conditions for local navigation and positioning purposes. In particular, responsiveness under curvilinear motions was investigated by means of an oscillating rotating table, allowing one to generate tangential and centripetal acceleration variations. The aim was to compare the responsiveness of the IMUs with respect to sinusoidal linear acceleration (generated by a typical vertical vibrating table, usually used in dynamic calibration procedures) and to sinusoidal curvilinear acceleration (generated by the oscillating rotating table proposed herein). To the authors’ best knowledge, this is the first time that a metrological comparison among linear, centripetal, and radial accelerations was investigated not only for IMU MEMS-based accelerometers but also for accelerometers in general.

Experimental evidence showed that the average values of the sensitivities along the three axes of the three IMUs under test, determined on the linear and rotating bench, are compatible, as normalized error results are systematically <1. This first evidence showed that, independently from the nature of acting motion along the three sensitive axes, the acceleration modulus is properly sensed, without spurious components that could provide incorrect output with respect to the local navigation and positioning tasks. Since the investigation was carried out at different frequencies and amplitudes, it was observed that no significant responsiveness differences were detected between the three different sensitive axes with respect to both radial and tangential directions, as well as among the three IMUs. The variability, with the standard deviation of values used to calculate the average sensitivity value (“Total”), for all the MEMS accelerometers did not exceed 1.4%.

Moreover, in the investigated frequency range, the average sensitivities determined for the *X*, *Y,* and *Z* axes are compatible with the scale factor declared by the manufacturer (i.e., 0.244 mg/LSB, corresponding to 418 D16 bit-signed/(m/s^2^)), although the scale factor provided at the manufacturer level does not have a traceability statement or an uncertainty budget. The evidence provided by this work allows one to link the data provided by the IMUs investigated along the traceability chain to SI, with a proper and well-defined uncertainty budget, allowing one to identify the suitable confidence levels for the safe and reliable management of local positioning and navigation in advanced smart mobility.

Future works, based on the experimental evidence presented here, will include the evaluation of the responsiveness of IMUs in vehicles, in real conditions, in order to verify technical performances in different operational scenarios. It is also planned to verify the long-term stability of the sensitivity measured in controlled laboratory conditions, after specific accelerated aging processes, in ranges of variable humidity, UV/solar radiations, salinity, pollutants, and other deteriorating or extreme conditions.

## Figures and Tables

**Table 1:** Average values of the sensitivities for the three axes of the three accelerometers under test, determined on the linear and rotating bench, and related expanded uncertainties (with *k* = 2 as coverage factor).

	BENCH	X	Y	Z
MEMS 1	curvilinear	423 ± 13	422 ± 11	425 ± 11
	linear	422 ± 13	419 ± 12	421 ± 11
MEMS 2	curvilinear	422 ± 9	423 ± 12	423 ± 9
	linear	422 ± 13	419 ± 11	419 ± 10
MEMS 3	curvilinear	423 ± 10	430 ± 11	426 ± 7
	linear	425 ± 22	429 ± 23	424 ± 17

**Table 2:** Normalized errors for sensitivities determined on the linear and rotating bench.

	X	Y	Z
MEMS 1	0.04	0.18	0.26
MEMS 2	0.05	0.25	0.31
MEMS 3	0.09	0.05	0.07

## Data Availability

The original contributions presented in the study are included in the article, further inquiries can be directed to the corresponding authors.

## References

[1] Venkat S., Fine C., Gonsalvez D. (2017). Faster, Smarter, Greener: The Future of the Car and Urban Mobility.

[2] Antonialli F., Martinesco A., Mira-Bonnardel S. (2022). Artificial intelligence as a determinant for reshaping the automotive industry and urban mobility services. Int. J. Automot. Technol..

[3] Grebe U.D., Hick H., Rothbart M., von Helmolt R., Armengaud E., Bajzek M., Kranabitl P. (2021). Challenges for future automotive mobility. Systems Engineering for Automotive Powertrain Development.

[4] Ahmed H.E., Sahandabadi S., Bhawya, Ahamed M.J. (2023). Application of MEMS Accelerometers in Dynamic Vibration Monitoring of a Vehicle. Micromachines.

[5] Calin I., Vlase S. (2023). Impact Attenuator Design for Improvement of Racing Car Drivers’ Safety. Symmetry.

[6] Manichandra B., Shiao Y. (2023). Advanced Driver Assistance Systems. The Future of Road Transportation.

[7] Wishart J., Chen Y., Como S., Kidambi N., Lu D., Yang Y. (2022). Fundamentals of Connected and Automated Vehicles.

[8] Musa A., Pipicelli M., Spano M., Tufano F., De Nola F., Di Blasio G., Toscano G. (2021). A review of model predictive controls applied to advanced driver-assistance systems. Energies.

[9] Sadruddin H., Atia M.M. (2019). Fusion of Digital Road Maps with Inertial Sensors and Satellite Navigation Systems Using Kalman Filter and Hidden Markov Models. Sens. Transducers.

[10] Fayyad J., Jaradat M.A., Gruyer D., Najjaran H. (2020). Deep learning sensor fusion for autonomous vehicle perception and localization: A review. Sensors.

[11] Elghazaly G., Frank R., Harvey S., Safko S. (2023). High-Definition Maps: Comprehensive Survey, Challenges and Future Perspectives. IEEE Open J. Intell. Transp. Syst..

[12] Feroz B., Mehmood A., Maryam H., Zeadally S., Maple C., Shah M.A. (2021). Vehicle-life interaction in fog-enabled smart connected and autonomous vehicles. IEEE Access.

[13] Arena F., Pau G., Severino A. (2020). An overview on the current status and future perspectives of smart cars. Infrastructures.

[14] Krasniqi X., Hajrizi E. (2016). Use of IoT technology to drive the automotive industry from connected to fully autonomous vehicles. IFAC-PapersOnLine.

[15] Khan A.R., Jamlos M.F., Osman N., Ishak M.I., Dzaharudin F., Yeow Y.K., Khairi K.A. (2022). DSRC technology in Vehicle-to-Vehicle (V2V) and Vehicle-to-Infrastructure (V2I) IoT system for Intelligent Transportation System (ITS): A review. Recent Trends in Mechatronics Towards Industry 4.0: Selected Articles from iM3F 2020.

[16] Gong C.S.A., Su C.H.S., Chen Y.H., Guu D.Y. (2022). How to implement automotive fault diagnosis using artificial intelligence scheme. Micromachines.

[17] Sehar N.U., Khalid O., Khan I.A., Rehman F., Fayyaz M.A., Ansari A.R., Nawaz R. (2023). Blockchain enabled data security in vehicular networks. Sci. Rep..

[18] Emami A., Sarvi M., Asadi Bagloee S. (2022). A review of the critical elements and development of real-world connected vehicle testbeds around the world. Transp. Let..

[19] Bazzan A.L., Klügl F. (2022). Introduction to Intelligent Systems in Traffic and Transportation.

[20] Kussl S., Wald A. (2022). Smart Mobility and its Implications for Road Infrastructure Provision: A Systematic Literature Review. Sustainability.

[21] Parekh D., Poddar N., Rajpurkar A., Chahal M., Kumar N., Joshi G.P., Cho W. (2022). A review on autonomous vehicles: Progress, methods and challenges. Electronics.

[22] Mihalj T., Li H., Babić D., Lex C., Jeudy M., Zovak G., Eichberger A. (2022). Road Infrastructure Challenges Faced by Automated Driving: A Review. Appl. Sci..

[23] Ahmed H.U., Huang Y., Lu P., Bridgelall R. (2022). Technology developments and impacts of connected and autonomous vehicles: An overview. Smart Cities.

[24] Greenblatt J.B., Shaheen S. (2015). Automated vehicles, on-demand mobility, and environmental impacts. Curr. Sustain./Renew. Energy Rep..

[25] Ru X., Gu N., Shang H., Zhang H. (2022). MEMS inertial sensor calibration technology: Current status and future trends. Micromachines.

[26] Podder I., Fischl T., Bub U. (2023). Artificial Intelligence Applications for MEMS-Based Sensors and Manufacturing Process Optimization. Telecom.

[27] Kourepenis A., Borenstein J., Connelly J., Elliott R., Ward P., Weinberg M. Performance of MEMS inertial sensors. Proceedings of the IEEE 1998 Position Location and Navigation Symposium.

[28] Jaffe R., Heiertz J., Ventresco S., Jaffe R., Heiertz J., Ventresco S. Testability of micromachined silicon rate sensors. Proceedings of the Guidance, Navigation, and Control Conference.

[29] Wendel J., Trommer G.F. Impact of Mechanical Vibrations on the Performance of Integrated Navigation Systems and On Optimal IMU Specification. Proceedings of the 57th Annual Meeting of The Institute of Navigation.

[30] De Pasquale G., Somà A. (2010). Reliability testing procedure for MEMS IMUs applied to vibrating environments. Sensors.

[31] Guo H., Yin Z., Cao D., Chen H., Lv C. (2018). A review of estimation for vehicle tire-road interactions toward automated driving. IEEE Trans. Syst. Man. Cybern..

[32] Capriglione D., Carratù M., Catelani M., Ciani L., Patrizi G., Singuaroli R., Pietrosanto A., Sommella P. (2020). Development of a Test Plan and a Testbed for Performance Analysis of MEMS-Based IMUs under Vibration Conditions. Measurement.

[33] Mohammed A.S., Amamou A., Ayevide F.K., Kelouwani S., Agbossou K., Zioui N. (2020). The Perception System of Intelligent Ground Vehicles in All Weather Conditions: A Systematic Literature Review. Sensors.

[34] Zhang Y., Carballo A., Yang H., Takeda K. (2023). Perception and Sensing for Autonomous Vehicles under Adverse Weather Conditions: A Survey. ISPRS J. Photogramm. Remote Sens..

[35] Yeong D.J., Velasco-Hernandez G., Barry J., Walsh J. (2021). Sensor and Sensor Fusion Technology in Autonomous Vehicles: A Review. Sensors.

[36] Wishart J., Como S., Forgione U., Weast J., Weston L., Smart A., Nicols G., Ramesh S. (2021). Literature Review of Verification and Validation Activities of Automated Driving Systems. SAE Int. J. Connect. Autom. Veh..

[37] Wu K.-W., Liao C.-C., Wu W.-F. Reliability and Safety Assessment of Automated Driving Systems: Review and Preview. Proceedings of the 2020 IEEE International Conference on Industrial Engineering and Engineering Management (IEEM).

[38] Schiavi A., Iacomussi P., Rossi L., Prato A., Mazzoleni F., Facello A., Genta G., Signoretti R. On the Trustworthiness of a Digital 3D MEMS Gyroscope Responsiveness to Dynamic Accelerations for ADAS Applications. Proceedings of the 2021 IEEE International Workshop on Metrology for Automotive (MetroAutomotive).

[39] Schiavi A., Prato A., Mazzoleni F., D’Emilia G., Gaspari A., Natale E. Calibration of Digital 3-Axis MEMS Accelerometers: A Double-Blind «multi-Bilateral» Comparison. Proceedings of the 2020 IEEE International Workshop on Metrology for Industry 4.0 & IoT.

[40] Prato A., Mazzoleni F., D’Emilia G., Gaspari A., Natale E., Schiavi A. (2021). Metrological Traceability of a Digital 3-Axis MEMS Accelerometers Sensor Network. Measurement.

[41] Iacomussi P., Schiavi A. (2022). Toward Metrological Trustworthiness for Automated and Connected Mobility. Electron. Imaging.

[42] Schiavi A., Mazzoleni F., Facello A., Prato A. Metrology for next Generation “Phygital Sensors”. Proceedings of the 2023 IEEE International Workshop on Metrology for Industry 4.0 & IoT (MetroInd4.0&IoT).

[43] Zhao S., Chen Y., Farrell J.A. (2016). High-Precision Vehicle Navigation in Urban Environments Using an MEM’s IMU and Single-Frequency GPS Receiver. Trans. Intell. Transport. Syst..

[44] Clausen P., Skaloud J., Gilliéron P.Y., Merminod B., Perakis H., Gikas V., Spyropoulou I. (2015). Position Accuracy with Redundant MEMS IMU for Road Applications. Eur. J. Navig..

[45] Schlögl M., Stütz R. (2019). Methodological Considerations with Data Uncertainty in Road Safety Analysis. Accid. Anal. Prev..

[46] D’Emilia G., Gaspari A., Natale E. (2016). Evaluation of Aspects Affecting Measurement of Three-Axis Accelerometers. Measurement.

[47] D’Emilia G., Gaspari A., Natale E. Calibration Test Bench for Three-Axis Accelerometers an Accurate and Low-Cost Proposal. Proceedings of the 2018 IEEE International Instrumentation and Measurement Technology Conference (I2MTC).

[48] Claybrook J., Kildare S. (2018). Autonomous Vehicles: No Driver…no Regulation?. Science.

[49] Biswas A., Wang H.-C. (2023). Autonomous Vehicles Enabled by the Integration of IoT, Edge Intelligence, 5G, and Blockchain. Sensors.

[50] iNEMO Inertial Module: Always-On 3D Accelerometer and 3D Gyroscope. https://www.st.com/resource/en/datasheet/lsm6dsr.pdf.

[51] iNemo Inertial Module Kit Based on ISM330DHCX. https://www.st.com/en/evaluation-tools/steval-mki210v1k.html.

[52] Prato A., Mazzoleni F., Schiavi A. (2020). Traceability of Digital 3-Axis MEMS Accelerometer: Simultaneous Determination of Main and Transverse Sensitivities in the Frequency Domain. Metrologia.

[53] Prato A., Mazzoleni F., Schiavi A. (2020). Evaluation and Correction of Systematic Effects in a Simultaneous 3-Axis Vibration Calibration System. Acta Imeko.

[54] (2003). Methods for the Calibration of Vibration and Shock Transducers, Part 21: Vibration Calibration by Comparison to a Reference Transducer.

[55] (2006). Methods for the Calibration of Vibration and Shock Transducers, Part 15: Primary Angular Vibration Calibration by Laser Interferometry.

[56] Gaitan M., Bautista I.M.L., Geist J. (2021). Reduction of Calibration Uncertainty Due to Mounting of Three-Axis Accelerometers Using the Intrinsic Properties Model. Metrologia.

[57] Gaitan M., Geist J. (2022). Calibration of Triaxial Accelerometers by Constant Rotation Rate in the Gravitational Field. Measurement.

[58] Geist J., Gaitan M. (2022). Type A Uncertainty Analysis Validation of Type B Analysis for Three-Axis Accelerometer Calibrations. Metrologia.

[59] Zhang M., Yan S., Deng Z., Chen P., Li Z., Fan J., Liu H., Liu J., Tu L. (2021). Cross-Coupling Coefficient Estimation of a Nano-g Accelerometer by Continuous Rotation Modulation on a Tilted Rate Table. IEEE Trans. Instrum. Meas..

[60] Schiavi A., Prato A., Mazzoleni F., Facello A., Kwitonda A. Metrological characterization of digital MEMS accelerometers in dynamic conditions: An investigation of linearity in amplitude and temperature effects. Proceedings of the 2022 IMEKO 5th TC22 Conference on Vibration Measurement 2022, Together with the 24th TC3 Conference on the Measurement of Force, Mass and Torque, the 14th TC5 Conference on the Measurement of Hardness, and the 6th TC16 Conference on Pressure and Vacuum Measurement.

[61] (2023). Conformity Assessment. General Requirements for the Competence of Proficiency Testing Providers.

